# Pre and post functional endoscopic sinus surgery nasal cavity volume assessment by acoustic rhinometry

**DOI:** 10.1016/S1808-8694(15)31003-X

**Published:** 2015-10-19

**Authors:** Rodrigo de Paula Santos, Walter Habermann, Thiemo Hofmann, Heinz Stammberger

**Affiliations:** a Master and Doctoral student, Head of the clinical rhinology department at the Sao Paulo Federal University - Paulista Medical School; b MD, Ph.D., Assistant physician of the Otorhinolaryngology Department at the Graz University, Austria; c MD, Assistant physician of the Otorhinolaryngology Department at the Graz University, Austria; d MD, Ph.D., Head of the Otorhinolaryngology Department at the Graz University, Austria. Sao Paulo Federal University/Paulista Medical School

**Keywords:** nasal polips, paranasal sinus, sinusitis

## Abstract

Acoustic rhinometry is an objective method to determine nasal cavity geometry. The technique is based on sound wave reflexion analysis in the nasal cavity, and determines crossectional areas as a function of distance as well as volume.

**Aim:**

The purpose of this study is to analyse nasal cavity volume changes caused by functional endoscopic sinus surgery (FESS) in adults with chronic rhinosinusitis by acoustic rhinometry, and to correlate these changes with improvements in the sensation of nasal obstruction.

**Material and Method:**

Forty patients aged from 18 to 73 years were prospectively evaluated between August and October 1999 at the Graz University Hospital, Austria. All patients were diagnosed with chronic rhinosinusitis, and undertook acoustic rhinometry before and after FESS.

**Scientific design:**

A clinical prospective study.

**Results:**

The nasal cavity total volume increased significantly after surgery. Nasal obstruction was improved in 88% of the patients, 20% with partial improvement and 68% with total improvement. There was no correlation between volume increase and improvement of the sensation of nasal obstruction.

**Conclusion:**

Total nasal cavity volume significantly increased after surgery; however, there was no correlation between volume increase and improvements of nasal obstruction. No significant pre or postoperative increase in total nasal cavity volume after decongestion were observed.

## INTRODUCTION

Functional endoscopic sinus surgery (FESS) was developed based on the pathophysiology theory of chronic rhinosinusitis proposed by Messerklinger, published in the 50s and 60s. In his original studies, this author used cadaver heads, impregnating the paranasal sinus mucus with a variety of substances. Using microscopes and endoscopes, he described the physiological pathways of paranasal sinus secretions. Based on these observations and correlations with findings in surgery on changes in mucociliary transport, Messerklinger was able to establish that the maxillary and frontal sinuses depend on their prechambers in the ethmoid and lateral nasal wall. Ventilation and drainage of these cavities are essential for normal nasal sinus function^1^.

FESS involves the opening of paranasal sinus prechambers aiming to restore drainage and ventilation. Messerklinger^2^ observed that eradication of the primary disease in the anterior ethmoid sinus through a limited endoscopic surgical procedure resulted in recovery of the mucosa of adjacent paranasal sinuses (frontal and maxillary) with no direct surgical manipulation of these areas. This is a conservative approach to surgical procedures on the nasal septum and turbinates.

Most papers published on this topic present subjective symptom relief results following FESS^3^, ^4^. The subjective perception patients have of their improvement is probably the best parameter to assess surgical efficiency, but objective methods are desirable to evaluate the impact of any therapeutic strategy.

There are few published papers studying objective assessment of the results of FESS. Most studies evaluate patients undergoing combined surgical procedures such as septoplasty and inferior turbinectomy.

Acoustic rhinometry (AR) is an objective method to measure the nasal cavity geometry^5^. The technique analyses the reflection of sound waves within the nose and measures the cross-sectional area of the nasal cavity in relation to the nares and defines the volume. This method was developed based on Jackson et al.'s^6^ studies on acoustic pulses to study the geometry of lower airways. This exam adds to the objective assessment of the nasal cavity, previously done with anterior rhinomanometry, which measures nasal air flow and resistance.

The aim of this paper is to study volume changes in the nasal cavity caused by FESS in adults with chronic rhinosinusitis with or without nasal polyposis using AR, and to correlate these alterations with clinical improvements and the sensation of nasal obstruction.

## MATERIAL AND METHODS

There were 40 patients (80 nasal cavities) included in this study, 21 women and 19 men, none of which had undergone nasal surgery. Patients were admitted consecutively at the Otorhinolaryngology ward of the Graz University Hospital in Austria, between August and October 1999, to undergo FESS. Age varied from 18 to 73 years. Patients with nasal septum deviation with an indication for septoplasty and patients with lower turbinate hypertrophy were excluded from the study.

All patients had symptoms of chronic rhinosinusitis for at least three months and had been treated with antibiotics, topical corticosteroids and antihistaminic drugs when indicated, with no significant improvement.

When medical treatment was not successful, patients would undergo rigid nasal endoscopy and computed tomography of the paranasal sinuses. The indication for surgery was based on the clinical history and the results of these two exams.

Rigid nasal endoscopy followed Messerklingler's^2^ methodology, assessing signs such as changes in the mucosa of the ethmoid infundibulum, the presence of pus in the rhinopharynx originated from the superior or middle meatus, and anatomical variants that led to narrowing or obstruction of the ostium-meatal complex, all of which are signs of chronic sinus disease.

Computed tomography was done to assess the degree of sinus involvement and as a guiding tool for surgery by revealing the anatomical relationship between the paranasal sinuses, the optic nerve, and the internal carotid artery^7^.

General anesthesia was used in all cases based on Stammberger's^8^ technique. Disease extension defined the type of surgical procedure, which included uncinectomy, anterior ethmoidectomy and perforation of the basal lamella of the middle turbinate in all cases. Posterior ethmoidectomy, sphenoidectomy, widening of the frontal recess and the maxillary sinus ostium were done as needed. No patient underwent surgery of the nasal septum or the lower turbinates.

AR was used as an objective evaluation method to separately measure nasal cavity volumes before and ten minutes after the use of a topical vasoconstrictor (two 0.5 mg/ml jets of oxymetazoline chloridrate nasal spray in each nare) pre and postoperatively. The vasoconstrictor was used to annul or minimize the influence of the physiological nasal cycle upon nasal cavity volume. Measurements were obtained one day before surgery and between four and eight weeks postoperatively, using the Rhinoklack - RK 1000tm (Stimotron Co., Wendelstein, Germany) device as internationally standardized^9^.

Out of 40 patients, 25 returned for a second evaluation. Before the second measurement, patients underwent postoperative endoscopy to clean the nasal cavities, removing clots that might interfere in exam results.

**Figure 1:**
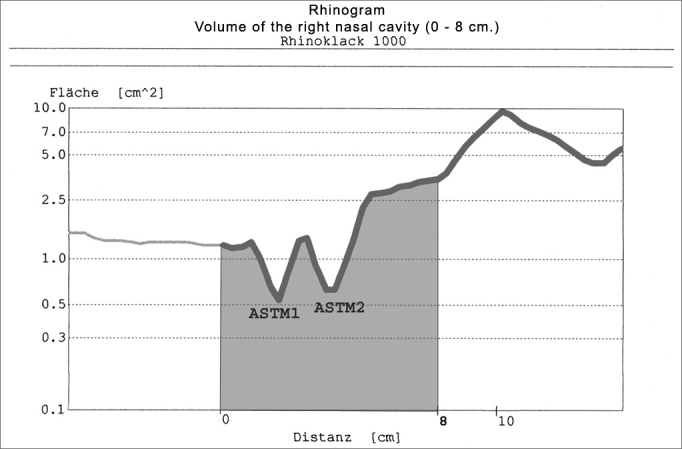
Rhinogram of the right nasal cavity, showing minimal cross-sectional areas 1 and 2 and volume (0 - 8 cm).

AR results are expressed in an “area x distance” graph on a semi-logarithmic scale, which is the rhinogram (Figure 1). On this chart the y-axis is the cross-sectional area (cm2) and the x-axis is the distance from the nasal adaptor into the nasal cavity (cm). The rhinogram of normal adults has two “dips”. They are known as the minimal cross-sectional areas 1 and 2. The volume between any two points in the nasal cavity is computer-calculated after these points are charted. We measured the volume between 0 and 8 cm distance from the nare^10^, ^11^. The total nasal volume was calculated as the sum of the right and left nasal cavities (Figure 1).

Data were analyzed statistically (Wilcoxon nonparametric test) comparing pre and post-vasoconstrictor total nasal cavity volumes before surgery, pre and post-vasoconstrictor total nasal cavity volumes after surgery and in the pre and postoperative periods following vasoconstrictor use to minimize the influence of the physiological nasal cycle.

Patients were inquired about changes in the sensation of nasal obstruction postoperatively compared to the preoperative period. We used a visual analog scale in which patients marked one of the following options: (-1) worsening of the sensation of nasal obstruction; (0) unchanged; (1) partial improvement; (2) full improvement. Nasal cavity volume changes produced by surgery were compared with the sensation of improvement from nasal obstruction. We used the Kruskal-Wallis nonparametric statistical test.

## RESULTS

Average nasal cavity volumes (right + left) before and after FESS and pre and post topical vasoconstrictor use are shown on Table 1. Nasal cavity volume increase following surgery was statistically significant.

**Table 1:** Average pre and post-vasoconstrictor nasal cavity volumes, before and after FESS, in cm3.

	Average preoperative volume of the nasal cavity (in cm3)	Average postoperative volume of the nasal cavity (in cm3)
Pre-vasoconstrictor	38,91	45,96
Post-vasoconstrictor	39,69	45,16

**Chart 1:**
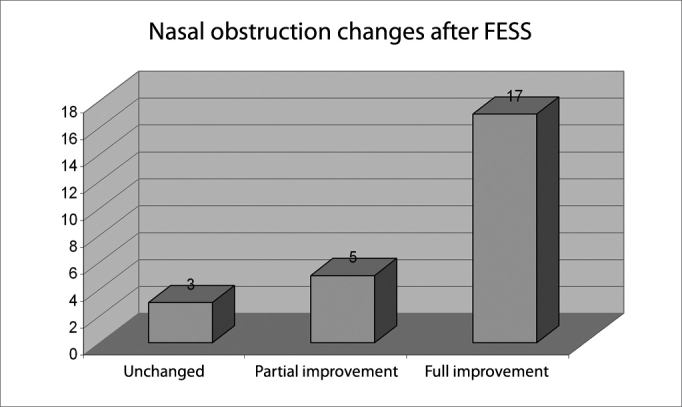

Results on the subjective complaint of nasal obstruction based on the visual analog scale are shown on Chart 1.

We used the Kruskal-Wallis test to study the different groups in the subjective analysis of the sensation of nasal obstruction groups (unchanged, partial improvement, full improvement), noting that the difference in nasal cavity volumes produced by surgery was not significantly different between the three groups (p = 0.311).

## DISCUSSION

FESS is currently the treatment of choice for chronic sinusitis that do not respond to medical treatment. Many studies with variable follow-up periods have focused on the subjective improvement of patients^1^, ^4^, ^12^, ^13^, ^14^, ^15^. AR, introduced by Hilberg et al.^5^, is an objective method to analyze the nasal cavity geometry by the use of sound waves. It is a simple non-invasive method requiring minimal patient cooperation. AR has been used in the pre and postoperative evaluation of rhinoplasties^16^, turbinoplasties and/or turbinectomies^17^, polypectomies^18^, adenoidectomies^19^, in assessing snorers and patients with obstructive sleep apnea syndrome^20^, choanal atresia^21^, subglottic stenosis^22^, and in children with chronic rhinitis^23^.

This study was made to obtain measurable data on FESS, due to the lack of objective evaluation data in literature on the result of nasal surgery. Results from various centers may thus be compared, and a possible relation between objective parameters and clinical improvement may be reached concerning nasal obstruction.

The total nasal cavity volume, obtained as the sum of the right and left nasal cavities, with an anterior-posterior distance of 0 to 8 cm, had an average value of 38.91cm^3^. This number is higher than Roithmann et al.'s^10^ and Lund & Scadding's^24^ values. This variation may be due to different inclusion criteria, ethnic differences, the type of device and the exam technique.

There was no significant difference between pre and post vasoconstrictor total nasal volumes both pre and postoperatively. There was an increase in nasal volume in normal individuals following the use of a vasoconstrictor, as reported in literature^25^. This effect was not observed in our study, possibly because our patients had chronic rhinosinusitis, where the mucosa does not behave normally.

The total nasal cavity volume was significantly increased following surgery (post-vasoconstriction), from 39.69cm^3^ to 45.16cm^3^ (p = 0.006), a 5.4cm^3^ average increase. This is supported by Lund & Scadding's^24^ and Hofmann et al.'s^26^ findings, who found average volume increases of 4.3cm^3^ and 4.1cm^3^ respectively. The nasal cavity volume measured after surgery is influenced by the maxillary and ethmoid sinuses. All patients in this analysis underwent anterior ethmoidectomy and, when necessary, posterior ethmoidectomy and amplification of the maxillary sinus ostium. The resulting volume increase is strongly correlated with these procedures.

Symptom improvement on the visual scale showed that no patient reported subjectively worse results for nasal obstruction, which was present in 100% of patients preoperatively. Three patients (12%) reported no change in nasal obstruction. There was improvement of nasal obstruction in 22 patients (88%), of which five patients (20%) reported partial improvement and 17 patients (68%) reported full improvement. These results are in agreement with numerous papers that assessed subjective improvements in patients following FESS, which reported improvement rates between 80% and 98%^1^, ^4^, ^12^, ^13^, ^14^, ^15^. Clinical improvement rates support the assumption that FESS operates on pathophysiological factors in chronic sinus disease^2^, ^3^.

In the various groups of nasal obstruction changes (no improvement, partial improvement and full improvement), we noted that there was no significant difference between nasal cavity volumes as a result of surgery in the three groups. Possibly this may be due to the small number of patients in each group, which reduced the efficacy of the statistical test (Kruskal-Wallis) to recognize any difference. Furthermore, the sensation of nasal permeability is more closely related to the minimal cross-sectional area than to the nasal cavity volume.

Various authors, however, have reported a low correlation between objective parameters obtained by acoustic rhinometry and rhinomanometry, and the subjective assessment of nasal obstruction^11^, ^26^, ^27^, ^28^, ^29^.

## CONCLUSION

Total nasal cavity measurement was increased postoperatively following FESS (5.4cm^3^ on average), which was statistically significant.

Acoustic rhinometry is useful to assess the FESS-related improvement of nasal obstruction, although there was not a linear relation between increased volume and the subjective improvement of nasal obstruction.

There was no significant difference between total nasal cavity volumes pre and post vasoconstrictor use both pre and postoperatively.
